# Communications, &c., Received

**Published:** 1859-09

**Authors:** 


*Communications, &c., Received.*—The publication of Dr. Dalton's interesting article in our Eclectic Department has made it necessary to defer the appearance of several communications which would otherwise have appeared this month.

We have received a letter from Dr. T. M. Bentley, of Jefferson, Wisconsin, detailing a case which is of great interest, and which we will publish in October, with some editorial remarks; also a communication from O. C. Tooker, M. D., of Greensburg, O.; one from Dr. Spencer, of Waterford, Pa.; and an obstetrical case from Dr. N. C. Husted, of this city.

We must also ask the indulgence of publishers for another month; when we will notice a number of the new works which are on our table.

Our Monthly Review of Parisian Medicine and Surgery is deficient this month, owing to a failure of the necessary journals to reach Dr. Mack. We are assured, however, by a letter from him, that he will be ready for the next number, and will embody the article which he has been compelled to omit this month.

---

